# Towards large-scale in free-standing graphene and N-graphene sheets

**DOI:** 10.1038/s41598-017-10810-3

**Published:** 2017-08-31

**Authors:** E. Tatarova, A. Dias, J. Henriques, M. Abrashev, N. Bundaleska, E. Kovacevic, N. Bundaleski, U. Cvelbar, E. Valcheva, B. Arnaudov, A. M. Botelho do Rego, A. M. Ferraria, J. Berndt, E. Felizardo, O. M. N. D. Teodoro, Th. Strunskus, L. L. Alves, B. Gonçalves

**Affiliations:** 1 Instituto de Plasmas e Fusão Nuclear, Instituto Superior Técnico, Universidade de Lisboa, Lisboa, 1049 Portugal; 2 Faculty of Physics, Sofia University, 1164 Sofia, Bulgaria; 3 GREMI UMR 7344 CNRS and Université d’Orléans, Orleans Cedex 2, France; 4 Departamento de Física, Faculdade de Ciências e Tecnologia, Universidade Nova de Lisboa, Lisboa, 2829-516 Portugal; 5 Department for Surface Engineering and Optoelectronics F4, Jozef Stefan Institute, Ljubljana, 1000 Slovenia; 6 Centro de Química-Física Molecular and IN, Instituto Superior Técnico, Universidade de Lisboa, Lisboa, 1049 Portugal; 7 CERN, Geneva, Switzerland; 8 Institute for Materials Science, Christian Albrechts Universitaet zu Kiel, Kiel, Germany

## Abstract

One of the greatest challenges in the commercialization of graphene and derivatives is production of high quality material in bulk quantities at low price and in a reproducible manner. The very limited control, or even lack of, over the synthesis process is one of the main problems of conventional approaches. Herein, we present a microwave plasma-enabled scalable route for continuous, large-scale fabrication of free-standing graphene and nitrogen doped graphene sheets. The method’s crucial advantage relies on harnessing unique plasma mechanisms to control the material and energy fluxes of the main building units at the atomic scale. By tailoring the high energy density plasma environment and complementarily applying *in situ* IR and soft UV radiation, a controllable selective synthesis of high quality graphene sheets at 2 mg/min yield with prescribed structural qualities was achieved. Raman spectroscopy, scanning electron microscopy, high resolution transmission electron microscopy, X-ray photoelectron spectroscopy and Near Edge X-ray-absorption fine-structure spectroscopy were used to probe the morphological, chemical and microstructural features of the produced material. The method described here is scalable and show a potential for controllable, large-scale fabrication of other graphene derivatives and promotes microwave plasmas as a competitive, green, and cost-effective alternative to presently used chemical methods.

## Introduction

Carbon-based 2D nanostructures have attracted an outstanding research interest due to their extraordinary properties^1, 2^ which make them desirable in numerous scientific and engineering disciplines. Research conducted both on fundamental and application levels of graphene and its derivatives led to an impressive number of scientific reports on the topic e.g., refs 3–6. The conjunction of intrinsic graphene properties with nitrogen functional groups translates in extraordinary electrochemical performances of nitrogen doped graphene, denominated as N-graphene^7, 8^. Preserving graphene’s large electric double-layer capacitance, N-graphene offers improved wettability and pseudocapacitance^7^. Lower resistivity and a specific capacitance of 282 Fg^−1^ at 1 Ag^−1^ was reported for N-graphene based supercapacitor’s electrode with excellent lifetime (>200,000 cycles), high power capability, and compatibility with flexible substrates^9^. By influencing atomic charge distribution N atoms create “activate sites” on the graphene scaffold that can participate in catalytic reaction directly, such as the oxygen reducing reaction in fuel cells or anchor metal nanoparticles used in the catalytic reaction^7^, making N-graphene a potential substitute for expensive metal catalysts. The use of N-doped graphene also improves the rate capabilities of graphene-based electrodes in lithium-ion and lithium-sulfur (Li-S) batteries. It exhibits strong-couple interactions for anchoring sulfur-containing species, thus achieving higher stability and reversibility when compared with pristine graphene^10, 11^, with an expected specific energy density of ~600 W∙h∙kg^−1^. Additional reviews on the progress of graphene/N-graphene and graphene based materials for energy storage and conversion devices are presented in^6, 12–14^.

One of the greatest challenges on the path towards graphene commercialization is the production of high quality material on a large scale at low cost and in a reproducible manner^15^. Graphene’s properties vary strongly as a function of its fabrication method and many of graphene and N-graphene’s outstanding properties are drastically dependent on its structural quality, i.e., sp^3^ carbons, defects, etc. Currently available commercial products (e.g.: https://graphene-supermarket.com/) exhibit a large variability of their physical properties, which substantially affects their usability.

Multiple processes have been reported for graphene synthesis, including mechanical exfoliation of natural or synthetic graphite^1, 3^, wet chemistry reduction techniques that employ graphite oxide (GO)^3, 16^, liquid-phase exfoliation^17^ and chemical vapour deposition (CVD)^18, 19^. Generally, these methods allow the synthesis of large-area graphene films and micron/submicron sized graphene sheets. CVD allows the production of large area sheets (currently of interest for low power graphene-based electronics and optical devices^4^), but its efficiency depends on the quality of the underlying polycrystalline metallic film (catalyst) and it requires multiple processing steps to obtain transferable sheets^3, 7, 18, 19^. Furthermore, CVD in the presence of carbon and nitrogen containing precursors (e.g. NH_3_, pyridine) is the most common direct method to prepare N-graphene^7, 8, 20^ on a nickel or copper substrate at a temperature of 1000 °C. It is worth noting that the presence of metal impurities can affect the real performance of the synthetized material. N-graphene sheets can also be produced via solvothermal methods^21^ and using an arc discharge with graphitic electrodes^22^. Post-synthesis methods such as thermal and chemical reduction of GO, along with simultaneous N-doping^23–25^ and plasma treatment of graphene^26, 27^ are also applied to produce N-graphene.

While promoting controllable, catalyst-free growth of graphene^28–30^, N-graphene like films^31–33^ on various types of substrates (e.g. metal (Ni, Cu, Al), dielectric (SiO_2_, quartz, etc) and 2D substrates (h-BN)) at lower temperatures, nearly all of the current plasma techniques involve plasma enhanced chemical vapor deposition (PECVD)^34^. The presence of energetic electrons in plasma environment boost the ionization, excitation and dissociation processes of carbon precursors at relatively low temperatures^35, 36^. The plasma systems comprise thermal and chemical reactor functions, as well as catalytic properties. Therefore, plasma assisted growth of nanostructures can be achieved without using catalysts at lower substrate temperatures due to the plasma’s unique ability to activate the surface, thus creating favourable conditions for nucleation and growth processes^37^. Unique aligned growth of vertical graphene on Ni foam and silicon support has been achieved^38, 39^. However, PECVD methods typically require low-pressure environments (<10 Torr) with the synthesis and growth of the structures occurring as in a conventional CVD proceed via reactions on the surface which depend on the substrate properties, often with low growth rates^34^. These methods struggle in producing high quality continuous and uniform graphene/N-graphene films on large areas.

Many applications (e.g.: supercapacitors, batteries, nanocomposites) require graphene nanosheets in the form of free-standing graphene/N-graphene sheets consisting of a few atomic monolayers^6, 11, 13^. Such self-standing graphene sheets are an alternative to surface-supported horizontal graphene because both surfaces can be utilized, while surface bound counterparts can effectively only use one, and allow the use of at least three open edges in applications. Some analysts foresee that free-standing sheets will dominate the market by 2025 (http://www.idtechex.com). The approaches commonly used to generate free standing graphene sheets are not focused on plasma methods, such as the ones based on colloids or thermal/chemical exfoliation of GO^16, 17^. While being recognized as a versatile and low-cost method for the preparation of graphene sheets at a relatively large scale, chemical reduction of GO (widely used for batteries/supercapacitors’ electrodes preparation) requires pre-production of GO from natural graphite via Hummers method and relies on the use of toxic chemical agents. The final products also exhibit a moderate electrical conductivity (i.e., presence of residues contamination, saturated sp^3^ bonds and bound oxygen groups)^16, 40–42^. To the best of our knowledge, only a couple of examples in the literature focus on the production of isolated free standing graphene sheets using plasmas at atmospheric pressure conditions. In the work of Dato^43^ free-standing graphene sheets synthesis using microwave plasma is reported without justification for the process that is simply referred as gas-phase synthesis. Moreover, the synthesized carbon material contains a large amount of amorphous carbon along with some individual graphene sheets as described in a related patent^44^. Selective synthesis of only high quality free-standing graphene sheets has been reported in our previous works but this was at the expense of quantity, i.e. at very low yield of about 0.01 mg/min^45–47^.

Here we intend to provide substantial evidence that microwave plasma technologies, and in particular these based on wave-driven plasmas, can be used as a competitive and disruptive alternative to chemical methods in the controlable fabrication of free-standing graphene sheets. The novelty of the plasma approach used is based on the process scale-up using large-scale configuration of microwave driven discharges and complementary engineering to control morphologies and structural qualities of targeted nanostructures. This is achieved via synergistic tailoring of the plasma environment and the “cold” outlet gas flow, where *in situ* infrared (IR) and ultraviolet (UV) radiation are applied. Besides Hirracane Cyclone system (http://www.acsystems.pt) is used for graphene sheet collection. With the patent describing the method recently issued, it becomes necessary to further investigate the scale-up of the process as well as a direct, single step N-graphene synthesis. The possibility to tune the density and energy of the building units, i.e., C_2_ radicals, C and N atoms, in the high energy-density plasma environment, which translates in an effective control over the energy and material fluxes towards growing nanostructures in the assembly zone of the reactor, is the most crucial advantage of this method. The plasma properties have been tailored to achieve selective synthesis of free-standing graphene/N-graphene sheets. Scanning electron microscopy (SEM) and high resolution transmission electron microscopy (HRTEM) as well as X-ray photoelectron spectroscopy (XPS) and Near Edge X-ray-absorption fine-structure (NEXAFS) spectroscopy^48^ were used to study the morphological, chemical and microstructural features of the synthesized nanostructures. The effect of N doping on the sample structure was studied by Raman and Fourier Transform Infrared (FT-IR) spectroscopies and XPS. Van der Pauw method was also applied to determine the electrical conductivity of the assembled graphene sheets^49^.

## The process

**Figure 1:**
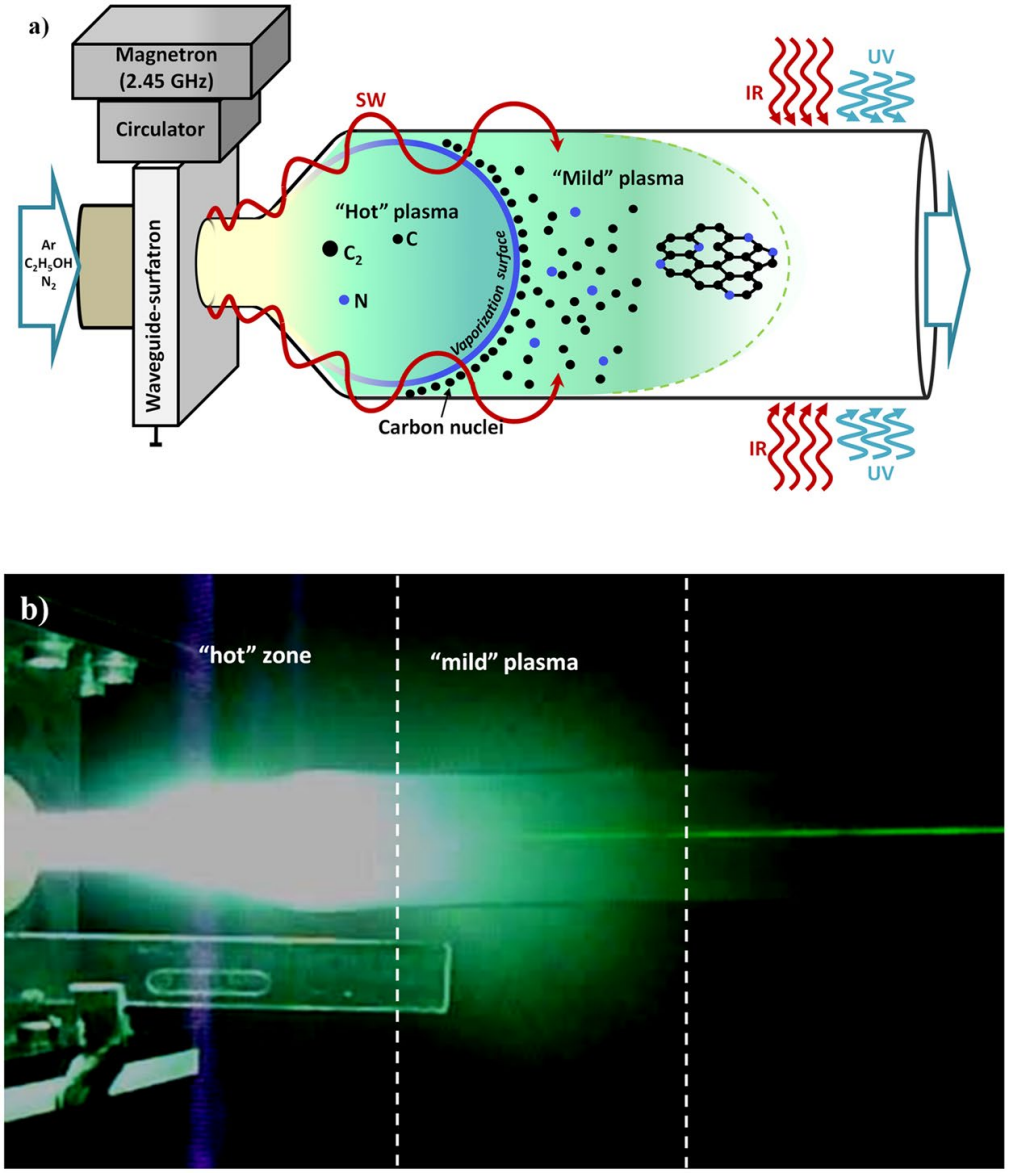
(**a**) Scheme of the process; (**b**) Photo of the plasma and “flowing” graphene sheets irradiated by laser beam.

A waveguide-surfatron based setup was used to create a surface wave (SW) induced microwave plasma at atmospheric pressure conditions (Supplementary material)^45, 46^. The microwave power is provided by a 2.45 GHz generator, whose output power was varied from 500 to 2000W. The generated plasma has an extended active zone outside the wave launcher, as seen in Fig. 1a, since it is sustained by the field of a travelling wave that simultaneously propagates and creates its own propagation structure. In this way, large microwave power densities can be delivered into the processing area and create high population densities of active species of interest. The discharge takes place inside a quartz tube inserted perpendicularly to the waveguide’s widest side and directed downstream. The tube contains a section with expanding radius and was designed to satisfy specific thermodynamic conditions (gas velocity, thermal fluxes, residence time etc.) into the “assembly zone” of the plasma reactor. The gas passing through the discharge results from the mixture of direct argon flow (background gas) and the combined flow of argon and carbon precursor, i.e., ethanol. To increase control over the synthesis process and improve the structural quality of the assembled flowing nanostructures both infrared and UV radiation (300–400 nm at 10 W) were applied in the gas-phase zone, where assembled nano-structures are dragged by the axial gas flow. A tornado type Hurricane Cyclone System collects the flowing sheets (http://www.acsystems.pt). A methanol glass container is connected to the exit of the Hurricane system to capture the smallest particles, capable to escape the system with the gas flow.

The plasma reactor can be considered as the assemblage of three different zones, as presented in Fig. 1. The first is the surface wave sustained discharge zone, including the zone inside the launcher and the extended “hot” plasma zone outside the launcher. Here, the wave power is absorbed primarily by plasma electrons, which transfer the power to heavy particles via elastic and inelastic collisions resulting in gas temperatures up to 4000 K. The gas temperature decreases slightly when moving away from the launcher up to about 15 cm before dropping considerably in the “mild” plasma zone (15–25 cm from the launcher). Carbon precursors are injected in the “hot” high energy density zone. Due to the collisions involving electrons and heavy particle along with intensive radical chemistry, decomposition processes of the injected carbonaceous molecules, i.e. C_2_H_5_OH, take place. The created radicals become precursors of chain reactions leading to creation of the main building blocks of carbon nanostructures, i.e. carbon atoms and C_2_ radicals^50^. It is worthy to note, that the assembling process requires very fast delivering and stacking of building units and equally fast supply of energy sufficient to overcome all the actual potential barriers. In the “mild” plasma zone, which includes also the “near” plasma afterglow, gas temperature drops from about 2000 K to 500 K. Note that the gas temperature changes also in radial direction, following closely a parabolic profile, due to the radial thermal losses. The transport of gas-phase carbon atoms/molecules into a colder zones through the so-called surface of vaporization (bold solid line in Fig. 1)^50^ results in transformation into solid carbon nuclei. Here, the schematic presentation of the isothermal plasma surface with constant temperature of about 1800 K is marked as “vaporization surface”. The transport of plasma generated carbon atoms/molecules into colder zones (outside of the vaporization boundary) of the reactor results in formation of solid carbon nuclei that are gradually withdrawn in the outlet plasma stream where kinetic processes of assembly and growth of “flowing” carbon nanostructures take place. Nitrogen atoms can be incorporated within the carbon nuclei via careful plasma tailoring and their density can be effectively controlled by the percentage of N_2_ flux in the gas mixture^51^. Given the fact that the nucleation and growth processes are determined by the interplay of kinetic and thermodynamic factors, the engineering of structural qualities of targeted nanostructures was achieved via synergistic tailoring of the “hot” plasma environment and thermodynamic conditions in the “mild” zone of the plasma reactor. Furthermore, the flowing structures can be a subject of infrared (*in situ* annealing) in the third, post-plasma zone and soft UV irradiation treatment, to “kick-off” the epoxy oxygen groups and sp^3^ carbons during the flight to a Hirracane Cyclone collecting system.

These three zones can be clearly distinguished in a photograph of the plasma reactor (Fig. 1b). The corresponding thermal map of the reactor, as obtained by a thermal imager, is shown below. The incident laser (532 nm, 1 W power) beam is scattered from the “flowing” graphene sheets, causing it to appear as a discontinuous green line on the photo. The scattered light from growing nanostructures appears in the “mild” plasma demonstrating that the graphene sheets are assembled and grown rapidly after crossing the “vaporization surface”. The sheets, being created in the plasma zone where ultrafast electrons rapidly settle on their surfaces, are negatively charged.

Using a cylindrical reactor with expanding radius allows the injection of large power density in the smaller radius section to achieve larger fluxes of building units (C_2_, C) with sufficiently high energy that will then flow towards the “mild” plasma zone, i.e. the assembly zone of the reactor. Once in the larger volume, the departure from the supersaturation conditions of the environment, prone to foster nanosheets assembling, is preserved^45, 46^. That is, the existence of a smaller amount of nuclei surpassing the critical size for nucleation and growth and conditions in which no supersaturation of species is possible promote the growth of existing nuclei rather than creation of new seeds. The reduction of Gibbs free energy of the system is behind the processes of nucleation and growth. This reduction of the Gibbs free energy of a saturated environment (e.g. plasma + solid phase) is proportional to the gas temperature. Lower gas temperatures, as well as larger densities of building units, foster supersaturation conditions in the environment and thus promote the synthesis of plenty nuclei and amorphous structures are more likely to be created. Contrarily, lower density of nuclei and higher temperatures lead to a deviation from supersaturation conditions, more prone to generate graphitic sheets. As expected, in the larger volume, the density of carbon nuclei decreases, and, as a result, the conditions favor the creation of planar nanostructures. Selective synthesis of graphene sheets only is achievable in a very narrow range of discharge operational conditions for the reactor design considered.

### High energy density plasma environment

**Figure 2:**
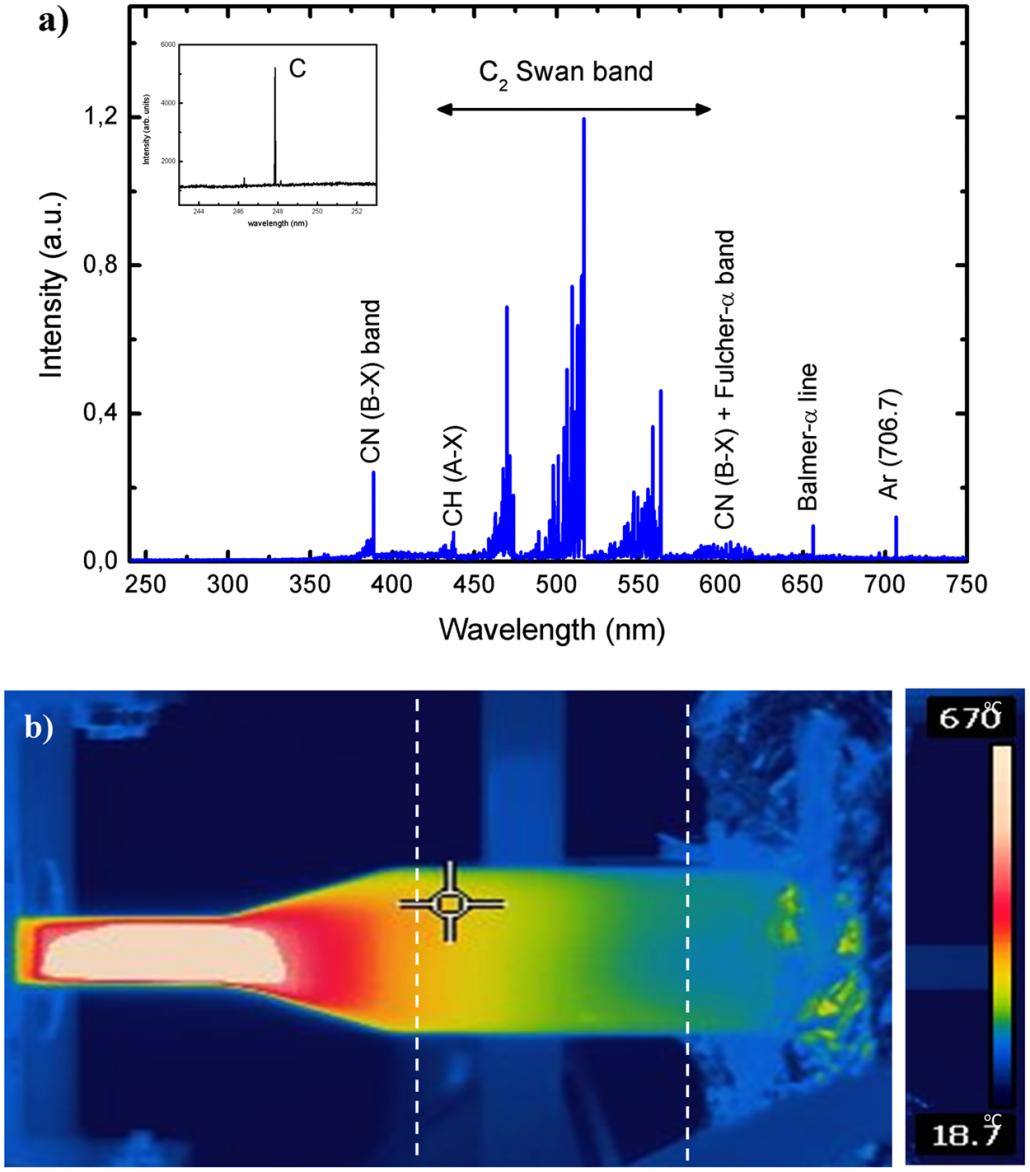
(**a**) Plasma emission spectrum (Q_Ar_ = 1200 sccm; Q_Et_ = 15 sccm, P = 2 kW); (**b**) 2D distribution of the temperature.

To identify the species of interest (C_2_, C) and to determine important plasma parameters such as the gas temperature in the discharge “hot” zone, plasma emission spectroscopy has been applied. Optical emission spectroscopy permits to identify the active carbon species in the plasma that are the “building units” for the synthesis and growth of the carbon nanostructures in the mild plasma stream. The detected argon/ethanol plasma emission spectra in the visible range (240–750 nm) are shown in Fig. 2(a). The emission spectrum of argon/ethanol plasma reveals the presence of new molecular and atomic species such as CN (violet system, B_2_Σ → X^2^Σ), C_2_ (Swan system, between 450–570 nm, A^3^Π_g_ → X^´3^Π_u_), C atoms (247.9 nm), the hydrogen Balmer-alpha line Hα (6563 Å) and several Ar lines. These species are formed as a result of ethanol decomposition. As mentioned, the experiments were carried out in the open atmosphere so that some nitrogen containing species also appear. As it can be seen in Fig. 2(a) strong plasma emissions of C_2_ molecular band, i.e. the Swan system, with heads band at 473.7, 516.5 and 558.6 nm were detected. The typical C_2_ emission is generated by the radiative decay of the ${{\rm{C}}}_{{\rm{2}}}^{\ast }{(A}_{{\rm{3}}}{{\rm{\Pi }}}_{{\rm{g}}})$ level. Due to the low energy threshold (E_ext_ = 2.4 eV), ground state C_2_ molecules can easily be excited to this level either by electron impact or by three body recombination processes involving carbon (C) and argon (Ar) atoms^50^. CN species are formed in a three-body recombination reaction: C + N + Ar → CN*(B^2^Σ) + Ar. The inset in the Fig. 2(a) demonstrates emission of carbon atoms at 247.9 nm.

The gas temperature in the “hot” plasma zone is a key parameter concerning ethanol decomposition. The collision rates in the plasma at atmospheric pressure are high enough to ensure local thermodynamic equilibrium. Therefore, the rotational temperature can be taken as a good approximation to the gas kinetic temperature. The emission band from the CN species corresponding to violet $\mathrm{CN}({{\rm{B}}}^{2}{{\rm{\Sigma }}}^{+}\to {{\rm{X}}}^{2}{{\rm{\Sigma }}}^{+})$ transitions was used to estimate the gas temperature in the “hot” plasma zone. An estimation of the gas temperature was obtained from measurements of the rotational distribution of the $\mathrm{CN}({{\rm{B}}}^{2}{{\rm{\Sigma }}}^{+}\to {{\rm{X}}}^{2}{{\rm{\Sigma }}}^{+})$ band in the 380–388.3 nm range. The experimental spectrum was fitted with a simulated one as obtained from LIFBASE software^52^. The estimations demonstrate that the rotational temperature measured at fixed background Ar flux (1200 sccm) and precursor fluxes in the range 15–30 sccm, remains nearly constant in the central “hot” zone with variation from about 4000 K to 3000 K. It should be mentioned, that this temperature is associated with the axis of the “hot” plasma column, since the radiation collected originates mainly from this region. Having a much lower value close to the wall, the gas temperature exhibits a sharp radial gradient. The 2D map of the temperature as detected by an infrared-sensitive thermal imager (FLIR camera) is shown in Fig. 2(b). As seen, the temperature demonstrates sharp decrease in the section with expanding radius and reaches nearly room temperature at about 30 cm away from the launcher.

## Results

### Graphene sheets synthesis

**Figure 3:**
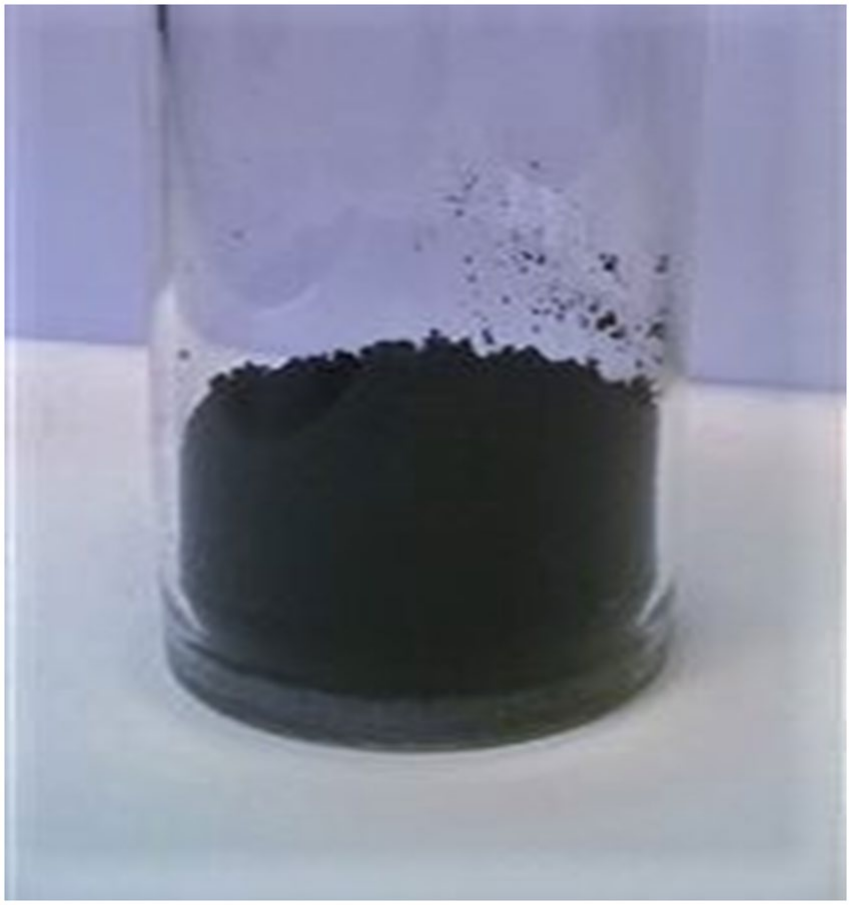
Graphene sheets as synthesized.

The range of operational conditions, i.e. background and precursor flow, microwave power etc. fostering selective synthesis (only sheets) is very limited. Outside of this range different carbon allotropes, i.e. amorphous carbon, 2D sheets etc, at the same time can be synthesized. Pure graphene sheets collected at background argon flow of 1200 sccm, power of 2 kW and ethanol flow of 30 sccm are produced at a rate of 2 mg/min. Figure 3 shows 1 g of graphene sheets as synthesized and collected by the Hurricane Cyclone system. The production costs of this black, light and fluffy material is estimated at 45 euros per gram, including electricity, cooling and used gases, which places the process very competitively considering the price of high quality graphene sheets products available in the market (e.g. https://graphene-supermarket.com).

**Figure 4:**
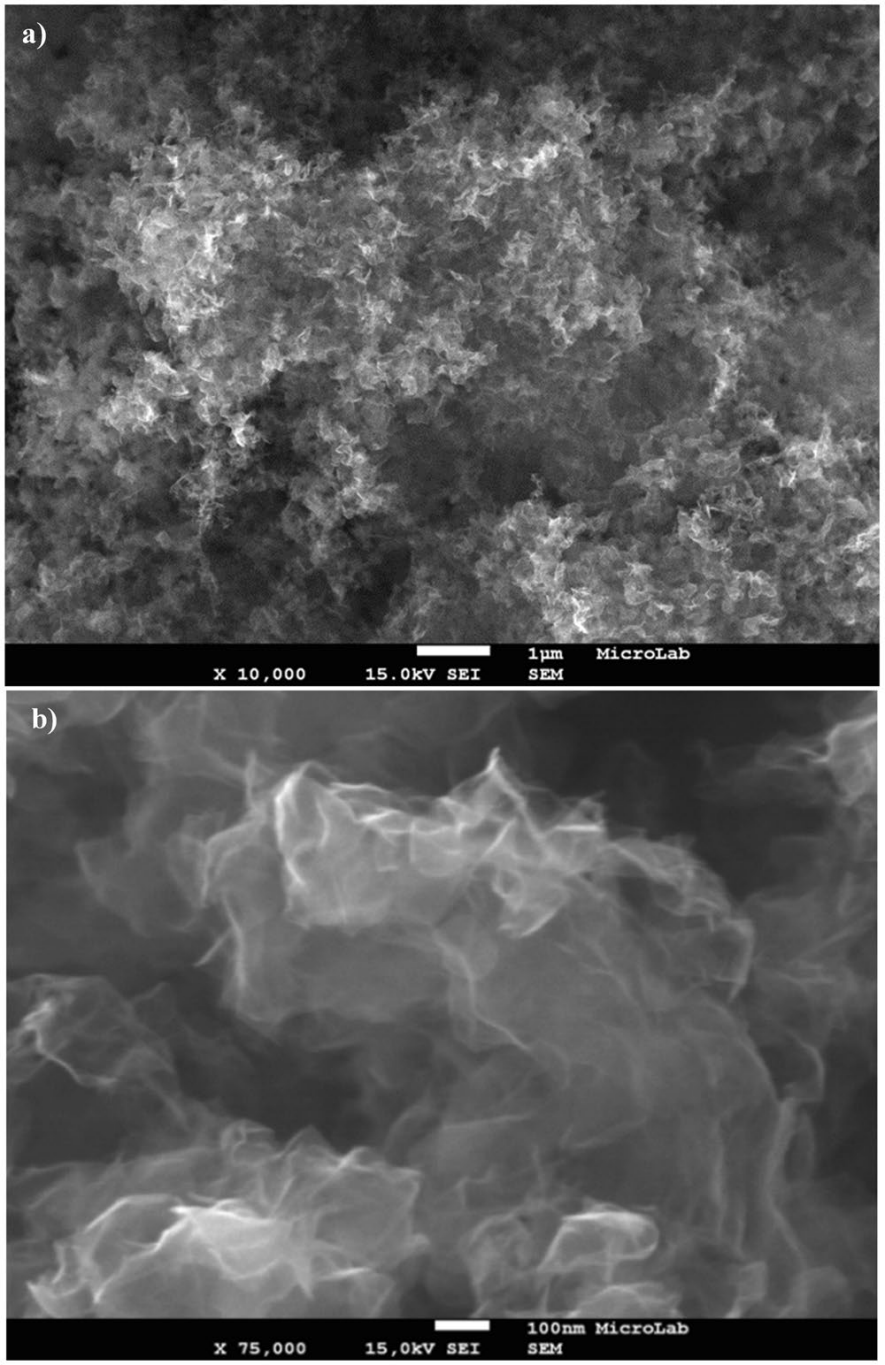
(**a**,**b**) Secondary electron images with different magnification of graphene sheets as synthesized.

In Fig. 4 SEM images of disordered sheets entangling each other are shown (Supplementary material). The sample contains only graphene sheets as evidenced by the low-magnification SEM image shown in Fig. 4(a). A characteristic curled/wavy morphology, consisting of a thin wrinkled paper-like structure is clearly seen. Higher magnifications, as shown in Fig. 4b, reveal ultrathin sheets with wavy structures.

**Figure 5:**
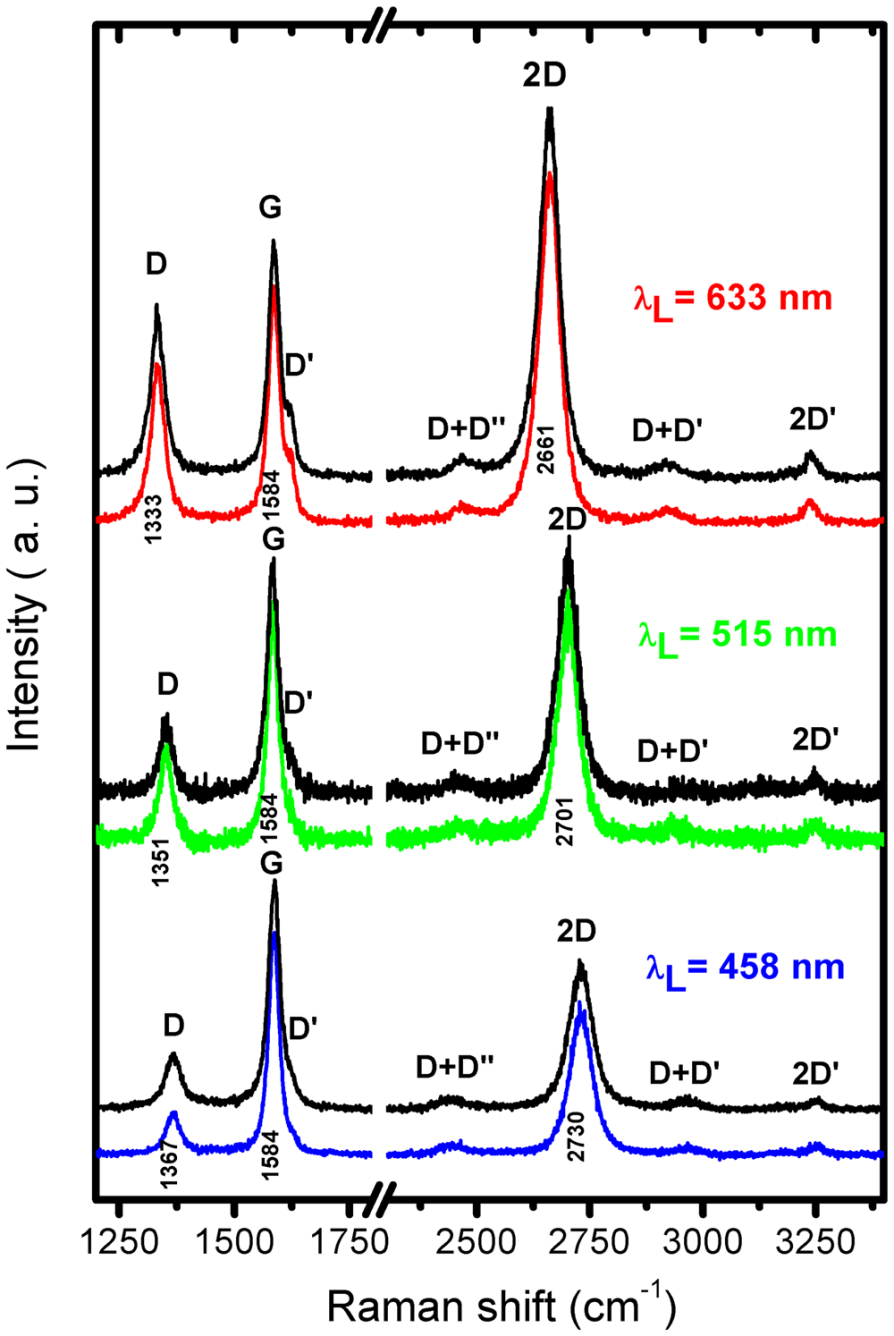
Raman spectra obtained with three excitation laser lines (633 nm, 515 nm, 458 nm) from two different spots of a single sample. Spectra were normalized to the intensity of the 1584 cm^−1^peak (denoted with G) and offset vertically for better viewing.

Raman spectroscopy is the most efficient way to provide a quick and easy structural and quality characterization of the samples. Typical Raman spectra of the synthesized carbon sheets produced at 2 kW microwave power, 1200 sccm Ar flow and precursor flow 30 sccm are shown in Fig. 5. In order to perform the Raman spectroscopy characterization, the synthesized nanostructures were freely suspended on a glass substrate and the Raman spectra from different randomly chosen spots were obtained and presented. The pairs of spectra, obtained with the same excitation, are practically identical, an evidence for the homogeneousness of the sample thus confirming that synthesis of only graphene sheets has been achieved. However, the relative intensity of all other lines and even their position are different and they strongly depend on the laser wavelength used. The line, denoted with D, changes its position from 1333 cm^−1^ (in λ_L_ = 633 nm spectra) up to 1367 cm^−1^ (in λ_L_ = 458 nm spectra). Its relative peak intensity (according to the G line (${I}_{{\rm{D}}}/({I}_{{\rm{G}}}$)) decreases from 0.70 to 0.20, respectively. The other strong line, denoted with 2D, changes its position from 2661 cm^−1^ (in λ_L_ = 633 nm spectra) up to 2730 cm^−1^ (in λ_L_ = 458 nm spectra). Its relative peak intensity (according to the G line (${I}_{2{\rm{D}}}/({I}_{{\rm{G}}}$)) decreases from 1.60 to 0.65, respectively. Although the primitive cell of graphene is very simple and it contains only two carbon atoms, the Raman spectra of graphene cannot be trivially interpreted. From a symmetry point of view, only one E_2g_ mode from the Г-point of the Brillouin zone is Raman-active^53^. This vibration corresponds to the G-line situated at 1584 cm^−1^ in all spectra. The appearance of the other lines is due to one-phonon forbidden scattering and/or two-phonon resonant scattering^54^. Their position depends on the excitation photon energy, whereas their intensity depends on the excitation photon energy and/or defects in the layer and the finite size of graphene flakes. The frequency dependence of the D and 2D lines was reported^54, 55^ and our spectra fully confirms it. The most prominent feature in the Raman spectrum of graphene is the 2D peak, whose position, shape and intensity are frequently used to distinguish between single-layer, bi-layer and multi-layer graphene. Taking into account the ratio between the 2D and G peak intensities and the full width at half maximum of the 2D-band (∼49 cm^−1^) the obtained results show that the samples contain graphene sheets with single or few mono-layers of carbon atoms^56^.

**Figure 6:**
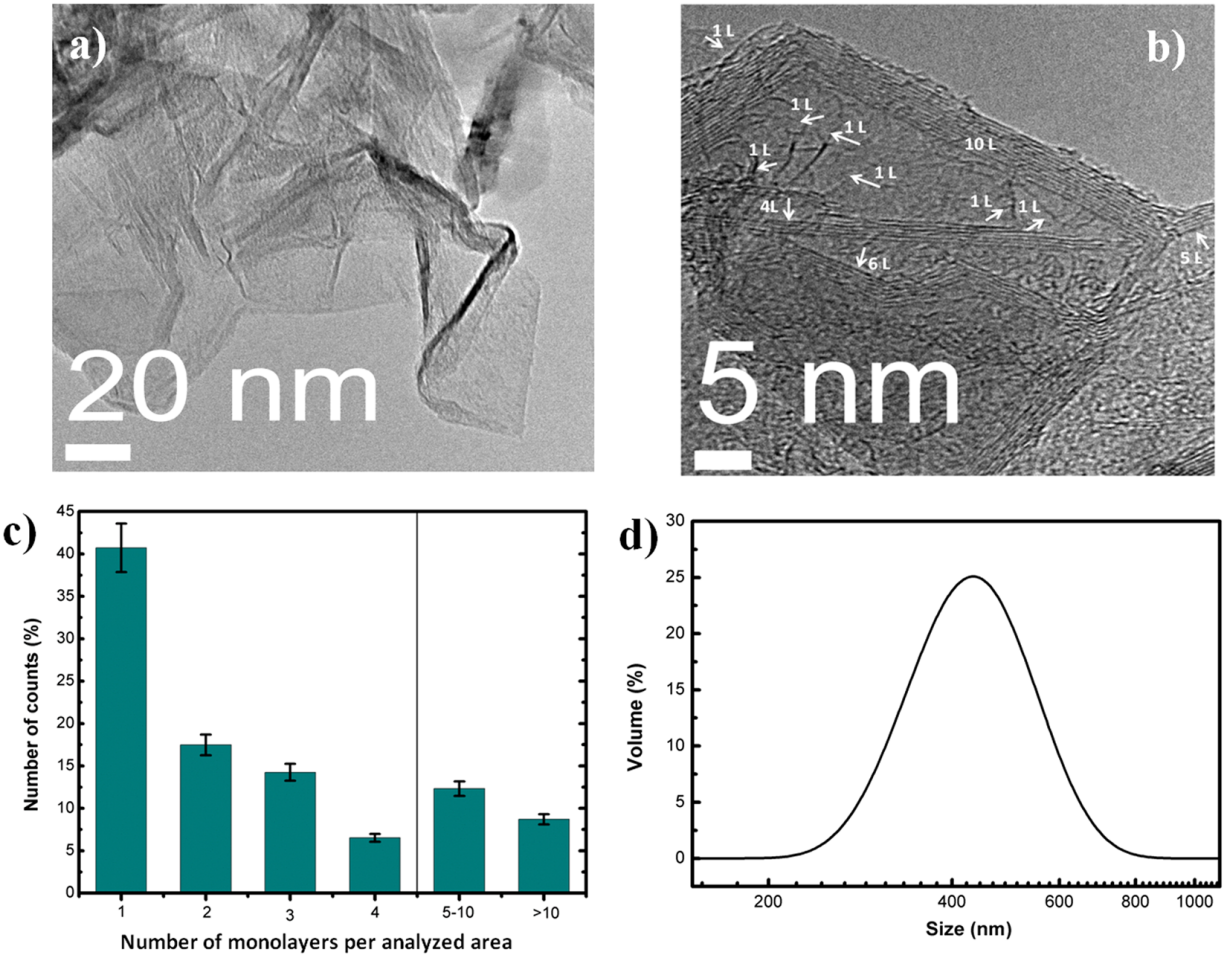
(**a**,**b**) HRTEM images with different magnification of freely suspended graphene. Monolayer (1 L) and multilayer (4 L, 6 L etc.) sheets are marked with arrows; (**c**) statistical distribution of the number of monolayers per analyzed graphene sample area of about 180 nm^2^; (**d**) size distribution of the sheets as obtained by Zetasizer Nano.

In addition to the recorded Raman spectra, the powder deposited on TEM grids allow the identification of monolayer and multilayer sheets (Fig. 6(a,b)). Less transparent areas can be attributed to the overlap of single or multilayer sheets while the darkest regions appear due to sheets crumpling. The evident upward curling at the edges of the individual sheets seen from the HRTEM image (Fig. 6(b)) may be due to internal stress in the few-layer graphene and these edges make it possible to evaluate their thickness. In a TEM image these edges appear as dark lines. Single (1 L) and multi-layers (up to 10 layers) graphene sheets can be easily distinguished. Moreover, highly ordered lattice fringes can be observed, indicating that the graphene sheets are well-crystallized. The interlayer distance is 3.6 Å and significantly larger than the 3.35 Å found in graphite.

A number of HRTEM images obtained from different randomly chosen graphene samples regions were analyzed. Considering that graphene sheets are crumpled, overlapped and/or entangled, the area ratio between monolayers graphene and the total graphene area is very difficult to determine. Therefore, we counting the number of monolayers/bilayers/multilayers from visual observation of HRTEM images. Analyzing a number of graphene areas (at 5 different positions), we produced the statistics presented in Fig. 6(c) that give the percentage of counts of monolayers sheets in all graphene sheets with an average surface area of ~180 nm^2^. Even though this is not the area ratio, the results give an indication about graphene sheets thicknesses and allow the calculation of the fraction of monolayers. For a set of randomly chosen samples, the percentage of counts of monolayers is about 40%. The investigated samples contained also bilayer, trilayer and higher order multilayer sheets. Approximately 15% of the counted multilayer sheets are composed by 5 to 10 monolayers while 10% contain 10 to 15 monolayers. The lateral size of the flakes is in the range 200–700 nm as seen from Fig. 6(d). Particle size distributions were evaluated by dynamic light scattering analysis with a Zetasizer Nano Series analyzer.

**Figure 7:**
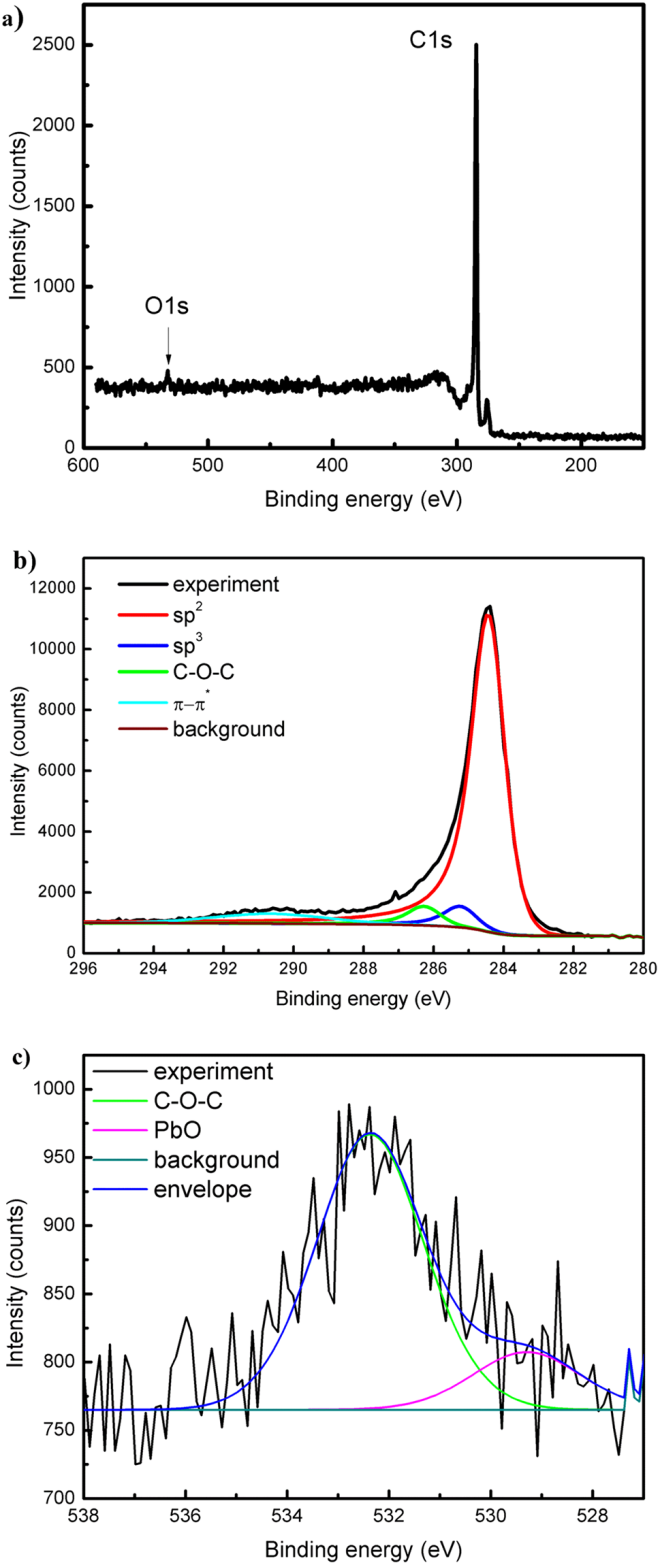
(**a**) XPS survey spectra of the sample obtained at P = 2 KW; Q_Ar_ = 1200 sccm; Q_Et_ = 26 sccm; T_wall  _ = 150 °C, UV radiation applied; (**b**) detailed C 1s region with corresponding fitting; (**c**) detailed O 1s region with corresponding fitting.

Furthermore, XPS and NEXAFS analysis have been performed to determine the elemental composition of the samples and to identify the chemical bonds (Supplementary material). The representative XPS spectrum in Fig. 7(a) shows a relatively small amount of oxygen in respect to the prominent carbon presence. The C 1s region and the corresponding energy loss features are shown in Fig. 7(b). The line was fitted to the superposition of four peaks: the first three peaks can be attributed to sp^2^ (284.4 eV), sp^3^ (285.2 eV) and C-O-C or C-OH (286.3 eV) bonds^57^, while the wide fourth peak represents π- π* shake up satellite (290.6 eV). Analysis of O 1s line fully support these findings, with the peak being well reproduced as the sum of aliphatic C-O-C bond (532.4 eV)^57^ and PbO (529.3 eV)^58^ contributions after taking into consideration different C 1s positions assumed for adventitious carbon (Fig. 7(c)). The later contribution originates from the oxidized lead surface, used to hold the graphene powder (Supplementary material).

The wall temperature (T_wall_ = 150 ^o^C) was measured at the reactor’s outlet gas zone (i.e., the region 15 to 25 cm away from the launcher) and it is indicative of the amount of IR irradiation that is simultaneously applied ***(***Supplementary material). The combination of UV irradiation and modest heating (i.e. 150 ^o^C) significantly increases sp^2^/sp^3^ ratio to about 15, while in the absence of both, annealing and UV irradiation, the same ratio is 10. This is probably due to the breaking of sp^3^ bonds by UV photons with an energy of about 3 eV (300–400 nm). The change in the amount of oxygen is, however, negligible (from 2.3% to 2.2%). At higher precursor fluxes (*Q*_*Et*_ = 36 sccm) and annealing temperature (260 ^o^C), keeping the other operational parameters constant (P = 2 KW; Q_Ar_ = 1200 sccm, UV irradiation applied), the relative amount of oxygen is two times smaller (~1.1%), whilst the sp^2^/sp^3^ ratio (15) is preserved. Under these conditions, defects are activated and annealed out by reopening the bonds and letting them reorganize to the perfect “crystalline” structure. This can be achieved only for a limited range of temperatures. Thus, the control/tuning of the thermodynamic conditions in the third (gas phase) zone of the reactor results in additional structural quality improvements of “flowing” graphene sheets.

**Figure 8:**
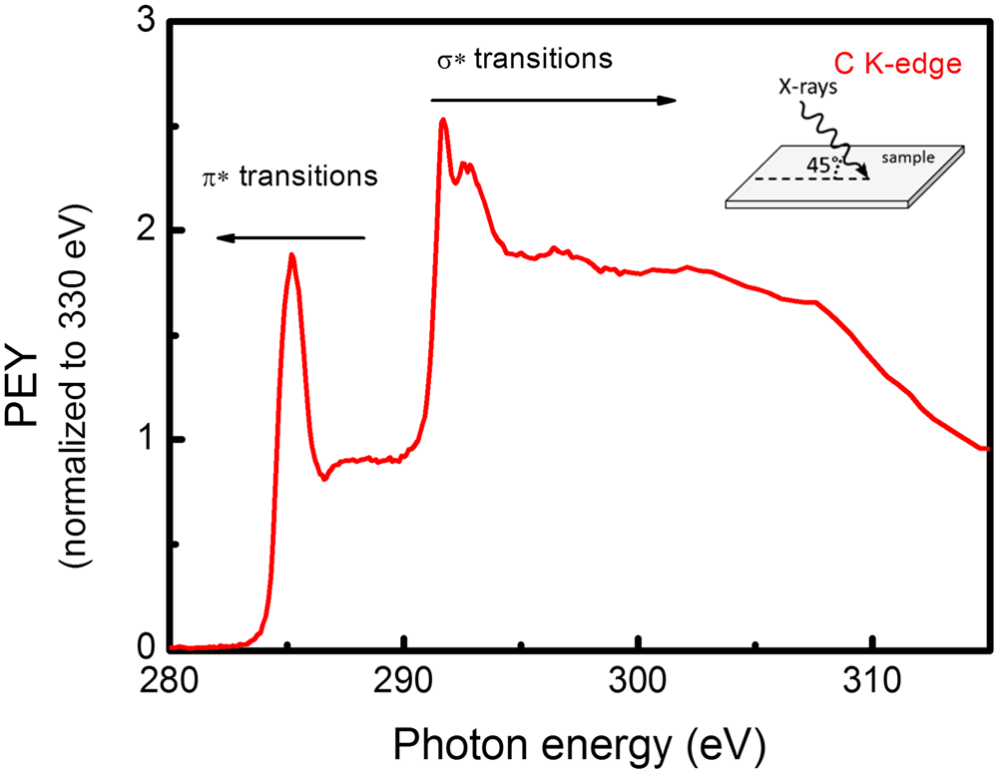
NEXAFS spectrum at C K edge of the graphene sheets synthesized at P = 2 kW, Q_Ar_ = 1200 sccm and Q_Et_ = 30 sccm and applied IR radiation (T_wall_ = 240^o^C). Normalized to the absorption jump, with post-edge intensity at 330.0 eV set to1^61^.

The nature of chemical intra molecular bonding has been further analyzed also by means of Near Edge X-ray-absorption fine-structure spectroscopy. Graphene powder was mechanically smeared onto different substrates. The substrates used for this specific measurement were intrinsic Si wafers. Spectra, as presented in Fig. 8 were obtained on the C K-edge, in the partial electron yield mode (PEY), which shows the very surface of the substrate^59^. A characteristic sharp C 1s → π* resonance is observable at ~285.1 eV^60, 61^. σ* resonances are observed at around 292 eV, with a sharp excitonic peak at ~291.7 eV, where π* and σ* refer to anti-bonding molecular orbitals, i.e. bands, of π and σ symmetry, respectively^60, 62^. Higher energy features are due to transitions towards higher-lying states of π or σ symmetry^63^. The lack of other peaks in-between is an indication of absence or low levels of oxygen and hydrogen. These results are in accordance with the previous XPS analysis, that shows about 2% of oxygen present on synthesized free standing graphene sheets.

To measure the electrical conductivity of the graphene sheets, 0.1 g of the graphene powder was pressed into a disc of 8 mm diameter and 1.2 mm thickness. The electrical conductivity was measured applying Van der Pauw method at room temperature^49^. The measurement geometry utilizes a four contact scheme where pinching point contacts are used, situated on the periphery of the tablet. This design provides measurement of the longitudinal conductivity as far as the current lines are parallel to the tablet surface. The current during the measurement was in the range 1–2 mA. Additionally current-voltage characteristics and reconfirmation of the Ohmic contact character were conducted for current values ranging from 0.5 to 3.5 mA. Several measurements were carried out and the average value is reported. The electrical conductivity of the graphene sheet was measured to be (3500 ± 350) S/m, which is much higher than the reported values of graphene sheets powder obtained by chemical methods^64^ and comparable with a reported value^65^. This could be explained with highly conductive sheets having multiple interfaces between them.

### *In situ* synthesis of N-graphene

**Figure 9:**
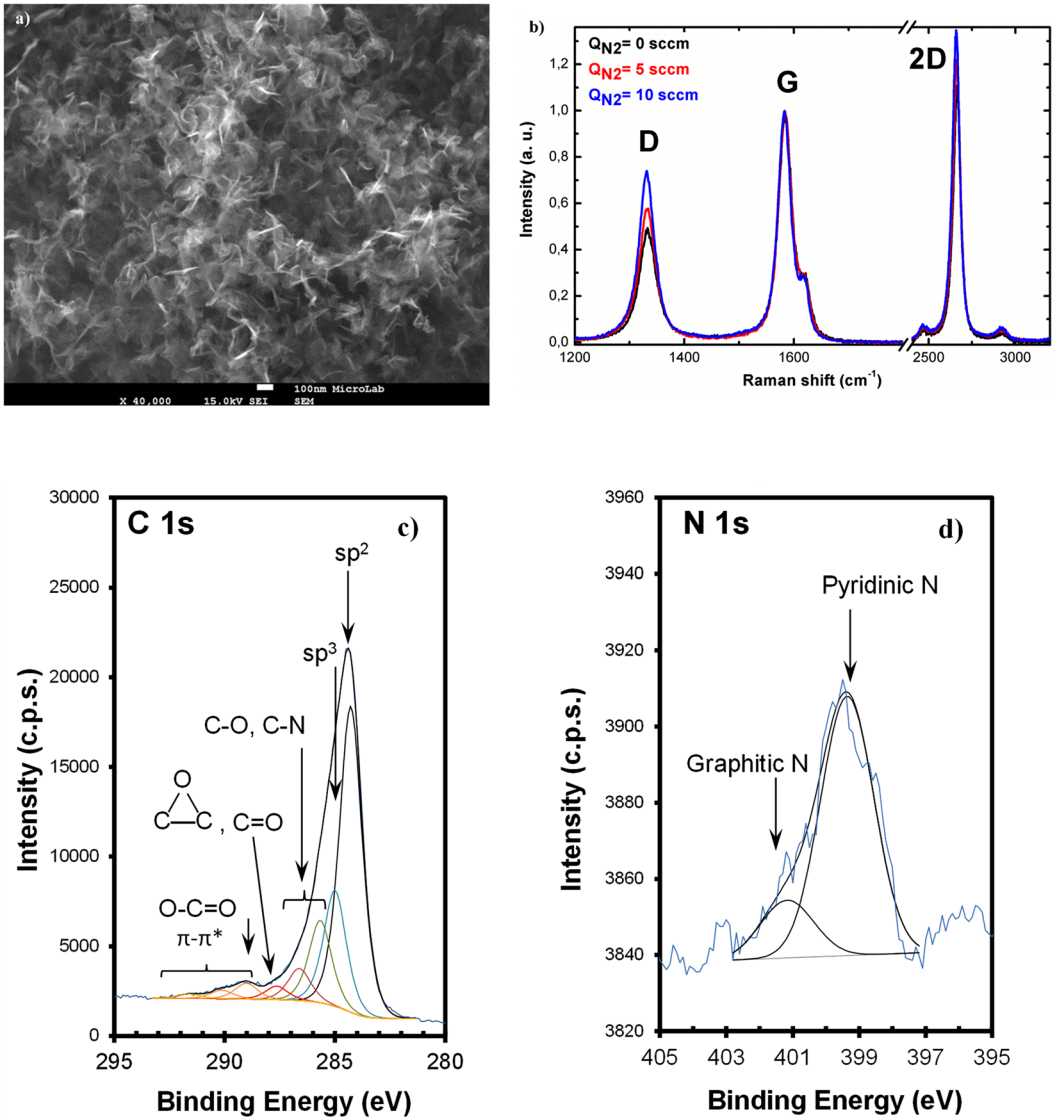
(**a**) SEM image of N-graphene sheets (Q_Ar_ = 1200 sccm; Q_Et_ = 55 sccm, Q_N2_ = 5 sccm). (**b**) Raman spectra obtained at different partial N_2_ flows (P = 2 kW, Q_Ar_ = 1200 sccm, Q_Et_ = 15 sccm); (**c**,**d**) Detailed C 1s and N 1s regions with corresponding fittings (P = 2 kW, Q_Ar_ = 1200 sccm, Q_Et_ = 15 sccm, Q_N2_ = 5 sccm).

In order to test the direct route for N-graphene synthesis as well as to demonstrate potential of the method, nitrogen gas flow (5–10 sccm) has been added to the ethanol flow. A small percentage of the N_2_ in respect to the background argon flow results in a high dissociation degree of N_2_ molecules therefore generating a significant amount of reactive nitrogen atoms (~10^22^ m^−3^) that can be incorporated into the growing carbon lattice structure. However, the dependence of N atoms density on gas mixture composition is to be noticed^51^. The rate of pure N-graphene sheets collected at a background argon flow of 1200 sccm, power of 2 kW, ethanol flow of 15 sccm and N_2_ flow of 5 sccm was about 0.5 mg/min. In the next Fig. 9(a), a moderate-magnification SEM image of disordered N-graphene sheets is shown. The sample contains only graphene-like sheets and indicates a more compact structure than the one of pure graphene sheets (Fig. 4(b)). Given the fact that N atoms incorporate the carbon lattice in graphitic, pyridinic, and pyrrolic configurations, it is conceivable that uneven distribution of charges in the different sheets promote the creation of attractive forces that increase their interactions. This phenomenon is worth further investigations and will be addressed in future research.

Furthermore, Fig. 9(b) shows the obtained Raman spectra for “pure” graphene sheets and its evolution with increasing N_2_ flow. Laser emitting at 633 nm has been used. As discussed, the three typical graphene peaks are attributed to the G band at ~1583 cm^−1^, 2D band at ~2658 cm^−1^, and D band at about ~1332 cm^−1^. The D band, i.e., “disorder” band, is related to a series of defects: bond-angle and bond-length disorder as well as hybridization that are caused by hetero atoms, i.e., nitrogen doping. The presented spectra are averaged over different locations. The most notable spectral change is the increase in the D band to G-band intensity ratio when N_2_ is included in the gas mixture. As seen from the figure, the D-peak intensity rises with the introduction of N_2_ gas. The nitrogen atoms constitute defects within the graphene lattice and consequently contribute to the D band intensity, yielding the observed increase of the D band to G-band intensity ratio.

Similarly, N-graphene sample chemical structures can be seen from XPS regions of C 1s and N 1s in Fig. 9(c,d). The main peak fitted in C 1s region is centered at 284.3 ± 0.1 eV which is assigned to sp^2^ carbon atoms bound to carbon or hydrogen atoms. The position of this peak was reported in our previous studies on free-standing graphene sheets synthesis induced by microwave plasma^46, 50^ and was obtained with no need of binding energy (BE) correction since no charge accumulation exists in this sample. Peaks centered at higher BE are assigned to sp^3^carbon atoms bound to other carbons or hydrogen atoms (285.0 eV), carbon bound to nitrogen or singly bound to oxygen (peaks at 285.7 and 286.6 eV), carbon in epoxide and/or carbonyl groups (287.6 eV) and carbon in carboxylate groups (289.0 eV)^57^. The spectral features detected at BE >290 eV are energy losses due to π-π* excitations typical of carbonaceous systems with delocalized π electrons such as graphene. The peak centered at 289 eV may be overlapping some of these features. Peaks found around 286 eV, which are, in a first approach, identified as C-N and C-O, are most probably superposing some aliphatic carbon atoms (***C***) bound to other carbons (***C***-C<) from more electronegative vicinities, for instance ***C*** *-*COO^−^ or ***C***-(C = O) (which can be found, roughly, between 285.3 and 286.2 eV)^57^. XPS confirms undoubtedly the doping of graphene by nitrogen atoms. The N 1s region was fitted with two peaks, one centered at a binding energy equal to 399.4 ± 0.2 eV assigned mainly to pyridinic nitrogen^57^, and a minor peak centered at 401.2 ± 0.2 eV, corresponding to graphitic nitrogen^66^. Pyrrolic nitrogen, which is likely to exist in these structures, must be close to 399.9 eV^67^, i.e. between the two fitted components. The shoulder found around 398.6 eV (not fitted) can be attributed also to pyridinic nitrogen in a slightly different chemical environment than the N 1s main peak (for instance in a more electron delocalized vicinity^68^. The quantitative analysis shows that the relative atomic concentration of nitrogen incorporated in the graphene scaffold is 0.2% for the conditions considered. The relative atomic concentration of incorporated oxygen is 8%.

**Figure 10:**
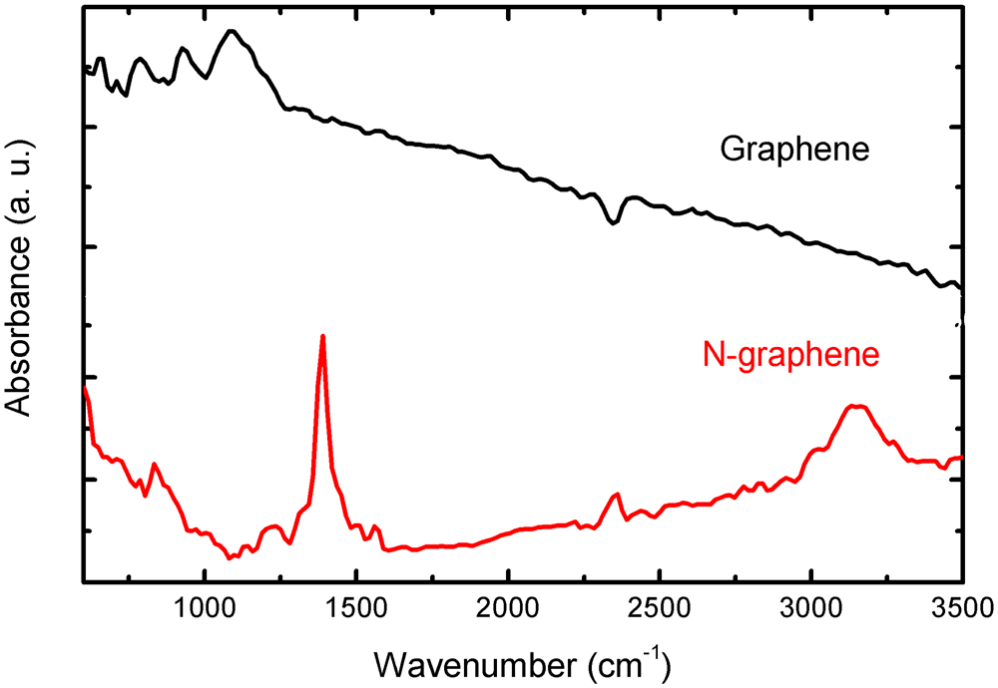
FT-IR spectra of graphene and N-graphene (P = 2 kW, Q_Ar_ = 1200 sccm, Q_Et_ = 15 sccm, Q_N2_ = 5 sccm).

FT-IR analysis was additionally employed to investigate the chemical bonding differences when graphene sheets are N doped (Fig. 10)^69^. The FT-IR spectrum of graphene sample is characterized by a broad peak at around 1100 cm−1–1220 cm^−1^, which can be attributed to C-O and C−O−C stretching bands, respectively, being in agreement with XPS results (see Fig. 7 (b))^70, 71^. In the IR spectrum of N-doped graphene the above peaks are not present, while several new bands appear. Few weak and broad peaks at 1220, 1340 (shoulder of a higher peak at 1390 cm^−1^), and 1550 cm^−1^ in the spectrum of N-graphene, can be attributed to C-N stretching vibrations and N–H band, respectively^70–72^. Additionally, the most intense peak at 1390 cm^−1^ (C–O–H stretching vibrations) is detected along with a wider band at around 3000 cm^−1^ (C-H)^73–76^.

### Lining-up free-standing sheets along the supporting surface

**Figure 11:**
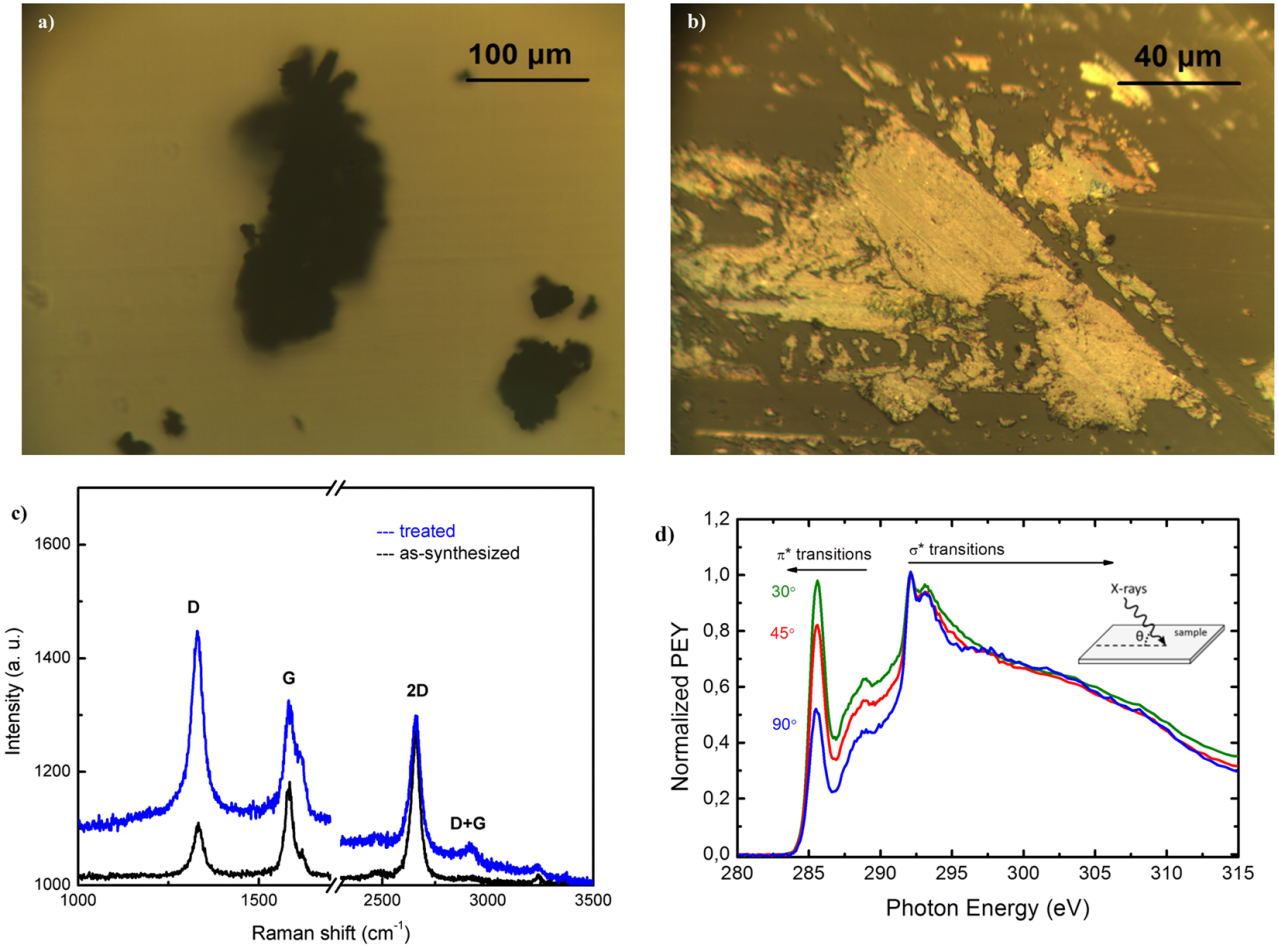
(**a**) Graphene sheets freely suspended on a glass substrate; (**b**) the same sheets spread over the glass with a metallic scalpel; (**c**) Raman spectra of the samples; (**d**) NEXAFS spectra of the graphene sheets spread over silicon substrate. Normalized to σ* resonance peak at $\sim$291.7 eV, indicated to observe angle dependence.

Processing graphene sheets is a delicate task since the charged sheets can reply in an ambiguous way during their treatment. Two photos of graphene sheets suspended on a glass substrate are shown in Fig. 11(a,b). The black graphene powder freely suspended on the glass substrate is shown in Fig. 11 (a), while the same powder after being spread over the glass using a metallic scalpel is shown in Fig. 11(b). As a result of this mechanical treatment, the graphene exhibits a yellow/golden color.

The corresponding Raman spectra are shown in Fig. 11(c). The spreading of graphene powder results in a sharp increase of the D-line at 1333 cm^−1^ and slight decrease of the G and 2D peaks. Moreover, a small frequency shift of the D-line of about 2 cm^−1^ towards smaller wavenumbers along with widening of the line is observed. The enhancement of the D-line, both absolute and relative to the G and 2D lines, may be simply related to the cutting-up of graphene sheets. However, the process may also force the parallel orientation of the sheets in respect to the supporting dielectric surface and lead to the formation of a network of electrically-oriented connected graphene sheets. In this case, the reduced G and 2D peak intensity could be linked to an increase in the conductivity of the whole layer and a corresponding decrease of the scattering volume.

To test this hypothesis, traces of graphene powder were mechanically smeared over silicon substrate (simple Si wafer as used for microelectronics) and analyzed by means of NEXAFS spectroscopy. Measurements were performed at different beam incident angles (θ) relative to the substrate surface as shown in Fig. 11. At the low-energy side a sharp resonance at $\sim$285.1 eV, corresponding to a C 1s - π* transition^60, 61, 77^, is observed, and second dominant feature, the double-structured resonance, is observed at around 292 eV, corresponding to C 1s - σ*. This double resonance comes from excitonic ($\sim$291.7 eV) and band-like contributions ($\sim$293.1 eV)^60, 62^. Considering the “fingerprint” region between 286 and 290 eV it is possible to identify some residual peaks not observed in previously shown spectrum in Fig. 8. In some studies, the origin of such peaks (specially broad shoulder) is connected with interlayer states in low symmetry regions of the Brillouin zone, as suggested by Fischer *et al*.^60^ (based on band calculations), from defects in the top most graphene layers^78^ or O-containing functional groups, resulting from oxidation (as it is most probably in our case).

NEXAFS is a powerful tool to observe molecular orientations due to the high linearly polarized nature of the synchrotron radiation, specifically for 1s → 2p excitations, where the product of the orbital symmetry axis and the electric dipole leads to simple intensity correlations between incidence angle and molecular orientation of the radiation^61, 77, 79^. There is a clear angular dependence of the C1s → π* resonance peak at 285.2 eV, with its intensity increasing towards the plateau regions and towards the intensity of excitons, as the incident angle relative to the supporting surface decreases. The differences are strongly correlated with orientation of the bonds relative to the surface and therefore support the existence of self-organization of the graphene sheets with respect to the surface of the substrate. In graphene sheets, the π bond deriving from p_z_ orbitals is oriented perpendicular to graphene plane (out-of-plane) and the σ bond is oriented along the intermolecular bonding axes (in-plane). Thus, as the angle between incident beam and surface increases out-of-plane excitation, i.e., π* resonance becomes weaker, and the ratio between σ* and π* resonances at 291.7 eV and 285.1 eV increases, as it is expected for highly oriented structures^61^ with a preferential orientation of the graphene sheets parallel to the substrate. It is to be noted that this alignment does not disappear over time or after functionalization by means of for example ammonia or nitrogen plasma.

## Conclusion

In summary, microwave-driven plasmas were successfully applied for the first time in the selective synthesis of high quality graphene and N-graphene sheets via a single step process at atmospheric pressure. A high level of control over oxygen functionalities and sp^2^/sp^3^ carbons ratio (~15) has been achieved. For a randomly chosen samples, the percentage of counts of monolayers in all graphene sheets is about 40%. The method is rapid, highly cost-efficient and environmentally friendly, since it does not require the use of catalysts and noxious chemicals. It is also customizable and versatile, allowing the synthesis of different types of 2D nanostructures (e.g. N-graphene,) in the same reactor. Furthermore, the high energy density of the generated plasma environment allows the use of gaseous, liquid or solid precursors. Moreover, self-organisation of the graphene sheets in respect to the supporting surface after mechanical treatment was observed.

To this end, our key enabling technology provides a rapid, single-step, cost-efficient and environmentally friendly method for selective synthesis of tailored graphene/N-graphene sheets at high yield and at atmospheric ambient. It is non-destructive, free of toxic chemicals, metal catalysts and substrates, and allows the use of carbon precursors in solid, liquid or gas state. The main advantage of our approach is the achivement of a very high and extremely controllable energy density in the plasma reactor, which allows effective control over the energy and material fluxes towards growing nanostructures at the atomic level via proper reactor design and tailoring of the plasma environment in a synergistic way. The ability to control the amount and localization of energy and matter delivered from the plasma bulk to the developing nano-structures is the key to achieve the desired morphological, structural and functional properties of targeted materials.

Further scale-up along with better understanding of nucleation/growth processes in a multiphase environment will certainly lead to a substantial increase of the yield and a reduction of the production costs. Besides, mastering will be focused on reducing the multilayer (5–10 single layers) sheets percentage below 1%, controllable increasing of N-doping and optimization of collecting system for extraction of nanoparticles with dimensions less than 20 nm, i.e. graphene quantum dots. The use of large-scale configurations of wave-driven discharges paves the way for further scale-up and a higher level of customization. The exclusive plasma mechanisms that rule the distributions of energy and matter at atomic scale endow the method significant potential for the synthesis of other 2D materials (e.g. boron-doped graphene, hBN, etc). The method has also a potential for producing unique graphene-metal nanocomposities with well-designed nano-architecture. The expanding pool of prospect applications will most certainly continue to drive forward the research in this cutting-edge field.

## Electronic supplementary material

Supplemetary Material
