# Supplementary material for: Towards large-scale in free-standing graphene and N-graphene sheets
Source: Sci Rep. 2017 Aug 31;7:10175. doi: 10.1038/s41598-017-10810-3 (PMC5579263; doi:10.1038/s41598-017-10810-3)
Supplement: Supplementary file 1 — Supplemetary Material [file 41598_2017_10810_MOESM1_ESM.doc]

**Towards large-scale in free-standing graphene and N-graphene sheets**

E. Tatarova^1^, A. Dias^1^, J. Henriques^1^, M. Abrashev^2^, N. Bundaleska^1^, E. Kovacevic^3^, N. Bundaleski^4^, U. Cvelbar^5^, E. Valcheva^2^, B. Arnaudov^2^, A M Botelho do Rego^6^, A. M. Ferraria^6^, J. Berndt^3^, E. Felizardo^7^, O.M.N.D. Teodoro^4^, Th. Strunskus^8^, L.L. Alves^1^, B. Gonçalves^1^

*^1^Instituto de Plasmas e Fusão Nuclear, Instituto Superior Técnico, Universidade de Lisboa, Lisboa-1049, Portugal*

*^2^Faculty of Physics, Sofia University, 1164 Sofia, Bulgaria*

*^3^* *GREMI UMR 7344 CNRS and Université d’Orléans, Orleans Cedex 2, France*

*^4^Departamento de Física, Faculdade de Ciências e Tecnologia, Universidade Nova de Lisboa, 2829-516 Portugal*

*^5^Department for Surface Engineering and Optoelectronics F4, Jozef Stefan Institute, Ljubljana 1000, Slovenia*

*^6^Centro de Química-Física Molecular and IN, Instituto Superior Técnico, UniversityofLisbon, Portugal*

*^7^CERN, Geneva,* [*Switzerland*](https://en.wikipedia.org/wiki/Switzerland)

*^8^Institute for Materials Science, Christian Albrechts Universitaet zu Kiel, Kiel, Germany*

**Supplementary Material**

**Experimental set-up**

A surfatron-based setup was used to create a surface wave induced microwave plasma at atmospheric pressure conditions^1^. The microwave power is provided by a 2.45 GHz generator (Sairem), whose output power was varied from 1000 to 2000W. The generator is connected to a waveguide (WR-340) system, which includes an isolator, directional couplers, a 3-stub tuner and a waveguide surfatron as the field applicator. The system is terminated by a movable short-circuit. The discharge takes place inside a quartz tube reactor, which is inserted downstream vertically and perpendicularly to the waveguide wider wall. The quartz reactor comprises two sections; a small one with internal and external radii of 0.75 cm and 0.9 cm, respectively, connected via conical section to the large tube with internal and external radii of 2.15 cm and 2.3 cm. A second quartz tube is used to introduce the vaporized precursor, *i.e.*, ethanol molecules, in the discharge zone. The background argon gas is injected into the discharge tube at flow rates varying from 1000 to 1500 sccm under laminar gas flow conditions. The precursor partial flux was varied in the range 5 to 30 sccm. Vaporization is performed by passing argon gas through a tank with liquid ethanol of 99.99% purity placed inside of sonication bath, with accurate control of the temperature. The total flow passing through the discharge consists of the direct argon flow passing through the large quartz tube plus the combined flows of the argon bubbling in the precursor liquid and the vaporized precursor, passing though the second quartz tube. Gas flow rates are controlled by a MKS247 Readout coupled to two MKS flow meters. The outlet gas stream temperature was actively controlled by an infrared irradiation of the wall. The system with network of IR lamps (length 20 cm) is placed immediately after the end of the discharge zone (in the range 15-30 cm away from the launcher). The wall temperature was monitored with FLIR thermal imager. The nanostructures were captured by a Hurricane Cyclone system followed by a methanol trap. The collected graphene powder was irradiated with UV lamps emitting in the range 300-400 nm at about 5 W.


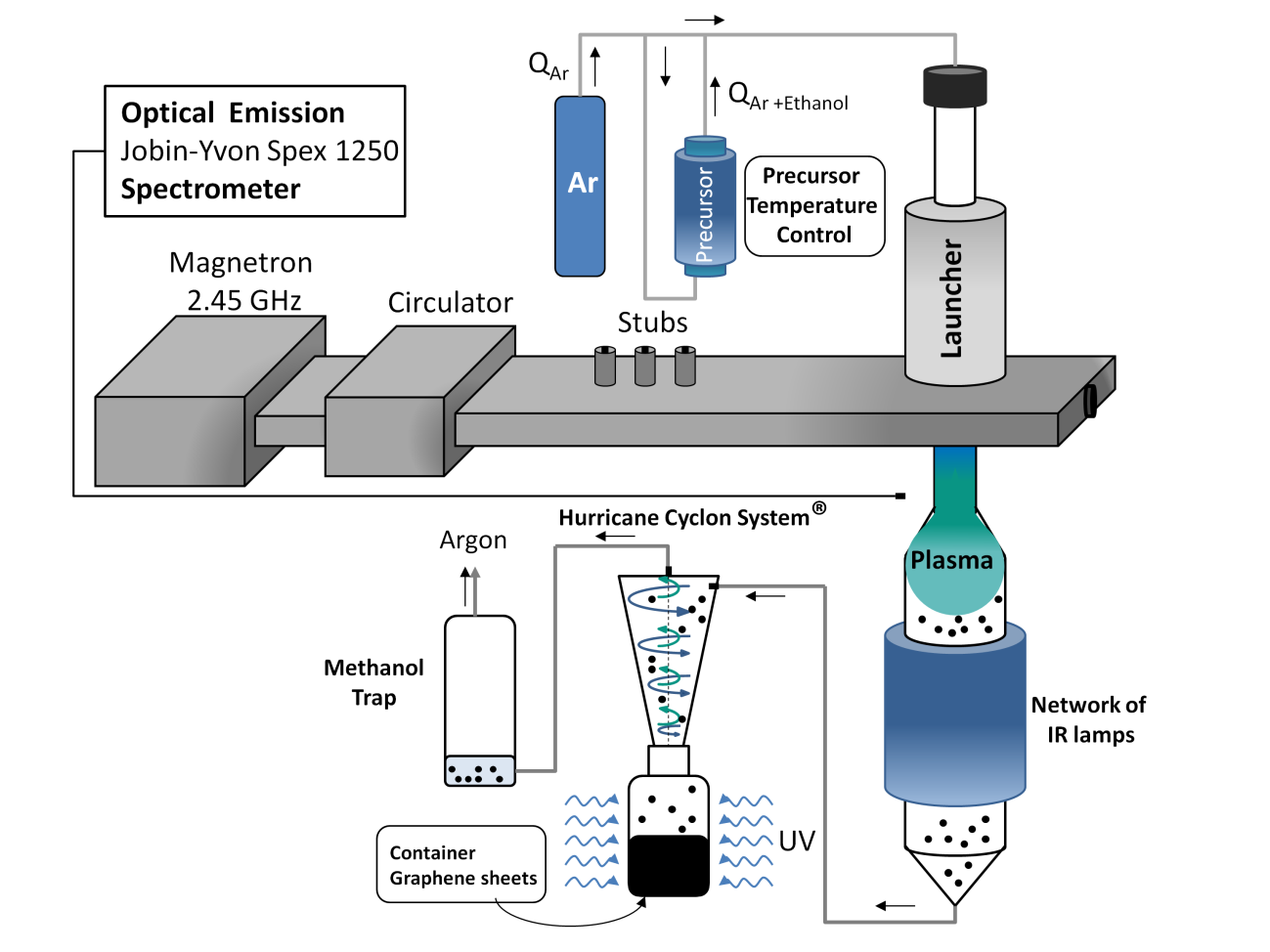


For gas sampling, a portion of the output gas stream from the plasma reactor (close to the late plasma afterglow region) was directed to an FT-IR Termo Nicolet 5700 spectrometer and the absorption spectra of species was detected in the wave number range 1000 – 4000 cm^-1^. The light emitted by the plasma was collected perpendicularly to the discharge tube by an imaging optical fiber and directed to the entrance of a Jobin-YvonSpex 1250 spectrometer (1200/2400 g/mm grating) equipped with a CCD camera. The cryogenic, back illuminated UV sensitive CCD camera has a 2048×512 matrix, featuring a 13.5 µm pixel-size, which provides high spectral resolution. A collimator located in front of the optical fiber defines the discharge volume (at the discharge axis) from where the plasma radiation is collected. The plasma emission spectra in the 230−750 nm range have been investigated.

**Characterization details**

**SEM** characterization of the samples has been performed by a JEOL, JSM-7001F field emission gun scanning electron microscope operating in secondary electron imaging mode (SEI) using 15kV accelerating voltage. The samples were deposited on a double-sided carbon tape mounted on an aluminium stub.

In order to perform the Raman spectroscopy characterization, the synthesized nanostructures were freely suspended on a glass substrate and the Raman spectra from different regions on the substrate were obtained using a LabRAM HR Visible (Horiba Jobin-Yvon) Raman spectrometer at 633 nm, 514 and 458 nm with 5 cm^-1^ spectral resolution and a laser spot size of 2 μm. Measurements were performed with a laser power *P_l_* = 0.054 mW to avoid overheating.

Graphene and N-graphene bonding situation was studied using a Fourier-Transform Infrared Termo Nicolet 5700 spectrometer in the wave number range 1000 - 4000 cm^-1^.

**HRTEM** The graphene flakes were placed directly as a solid powder onto a copper grid for transmission electron microscopy (TEM). The sheets were then characterized by a high-resolution TEM JEOL JEM-2010F, operating at 200 kV accelerating voltage.

**X-ray photoelectron spectroscopy** XPS spectra of N-graphene were obtained with a KRATOS XSAM800 X-ray spectrometer with double anode, operating in Fixed Analyzer Transmission mode, with analyser pass energy of 20 eV and non-monochromatic X-radiation with a power of 120 W (12 kV×10 mA). Samples were analysed at room temperature, at UHV pressure around 10^−7^ Pa and a take-off angle set to 90º. Graphene sheets were pealed from the filters using the XPS sample holder with double-side tape. The spectra were collected with 0.1 eV steps, using a Sun SPARC Station 4 with Vision software (Kratos). X-ray source satellites were subtracted. Shirley backgrounds and Gaussian/Lorentzian line shapes were fitted using XPS Peak 4.1 (freeware). No flood gun was used for charge compensation. Binding energies (BE) were corrected taking as a reference the binding energy of sp^2^ carbons.

XPS characterization of pure graphene sheets was carried out on a VSW XPS system with the Class 100 energy analyzer being a part of an experimental setup (Multitecnica) assembled for surface investigation. The samples were pressed onto 1 mm thin lead plates, in order to provide both mechanical support and electrical contact. The survey spectra were taken in a fixed analyzer transmission mode with the pass energy of 44 eV, i.e., FAT 44, while the detailed spectra of characteristic lines were taken in FAT 22 mode. The analysis has been performed using the non-monochromatic Mg K_α_ line (photon energy of 1253.6 eV). The carbon line was fitted to Doniach-Šunjić profile convoluted with pseudo-Voigt GL(70) shape^2-4^. The applied peak fitting model was established from the previous measurements of freshly cleaved highly oriented pyrolytic graphite. The oxygen line was fitted to pseudo-Voigt GL(30) line shapes. For the energy axis calibration, Ag (110) and polycrystalline Au samples (previously cleaned by ion sputtering) were used. The energy was calibrated to the peak position of Ag 3d_5/2_ (binding energy of 368.22 eV) and Au 4f_7/2_ (binding energy of 83.96 eV) lines. The samples were analyzed *ex situ* without cleaning, in order to avoid bond breaking.

**NEXAFS** Near Edge X-ray-absorption fine-structure (NEXAFS) spectroscopy was performed on the samples using the HE-SGM beam line at BESSY II storage ring (PREVAC end station provided by Professor Ch. Wöll). NEXAFS measurements were performed considering different incident angles (20-90°) relative to the substrate surface. The data was collected in respect to the C K-edge to characterize the carbon chemistry of the produced free-standing graphene, in the partial electron yield (PEY) mode (few nm depth), using a home built double channel plate detector. The energy resolution was ≈0.40 eV. The raw NEXAFS spectra were corrected for the beam line transmission by division through a spectrum of a clean, freshly sputtered Au sample. Alignment of the energy scale was accomplished by using an I0 feature referenced to a C 1s → π* resonance at 284.9 eV from a fresh surface of a graphite foil standard sample^5^.

**References**

1. M. Moisan and Z. Zakrzewski, J. Phys. D: Appl. Phys. **24**, 1025 (1991).

2. Doniach, S.; Sunjic, M. *J. Phys. C1970, 3,* 285.

3. Kidambi, P.R. *Nano Lett.* 2013, *13,* 4769.

4. Dias, A.; Bundaleski, N.; Tatarova, E.; Dias, F.M.; Abrashev, M.; Cvelbar, U.; Teodoro, O.M.N.D.; Henriques, J. *J. Phys. D: Appl. Phys.* 2016, *49,* 055307.

5. Schmidt C., Breuer T., Wippermann S., Schmidt W.G. and Witte G. *“Substrate Induced Thermal Decomposition of Perfluoro-Pentacene Thin Films on the Coinage Metals”* J. Phys. Chem. C**116** (45) (2012) 24098–24106.
